# A wild-type mouse-based model for the regression of inflammation in atherosclerosis

**DOI:** 10.1371/journal.pone.0173975

**Published:** 2017-03-14

**Authors:** Michael Peled, Hitoo Nishi, Ada Weinstock, Tessa J. Barrett, Felix Zhou, Alexandra Quezada, Edward A. Fisher

**Affiliations:** Department of Medicine, Leon H. Charney Division of Cardiology, and the Marc and Ruti Bell Program in Vascular Biology, New York University School of Medicine, New York, New York, United States of America; Ludwig-Maximilians-Universitat Munchen, GERMANY

## Abstract

Atherosclerosis can be induced by the injection of a gain-of-function mutant of proprotein convertase subtilisin/kexin type 9 (*PCSK9*)–encoding adeno-associated viral vector (AAVmPCSK9), avoiding the need for knockout mice models, such as low-density lipoprotein receptor deficient mice. As regression of atherosclerosis is a crucial therapeutic goal, we aimed to establish a regression model based on AAVmPCSK9, which will eliminate the need for germ-line genetic modifications. C57BL6/J mice were injected with AAVmPCSK9 and were fed with Western diet for 16 weeks, followed by reversal of hyperlipidemia by a diet switch to chow and treatment with a microsomal triglyceride transfer protein inhibitor (MTPi). Sixteen weeks following AAVmPCSK9 injection, mice had advanced atherosclerotic lesions in the aortic root. Surprisingly, diet switch to chow alone reversed hyperlipidemia to near normal levels, and the addition of MTPi completely normalized hyperlipidemia. A six week reversal of hyperlipidemia, either by diet switch alone or by diet switch and MTPi treatment, was accompanied by regression of atherosclerosis as defined by a significant decrease of macrophages in the atherosclerotic plaques, compared to baseline. Thus, we have established an atherosclerosis regression model that is independent of the genetic background.

## Introduction

Induction of hypercholesterolemia and atherosclerosis in animal models has provided critical insights in the pathogenesis of atherosclerosis [1, 2]. Though most studies are focused on the progression of atherosclerosis and its prevention, the more common clinical setting is of a middle-aged patient that already has a substantial burden of atherosclerosis, making the ideal goal of therapy the induction of atherosclerosis regression.

Atherosclerotic plaque regression can be determined in several ways, including reduction in plaque size, plaque cholesterol content, or a decreased inflammatory component as assessed by a reduction in plaque macrophage number/percentage.[3]. In this report, we have considered atherosclerosis regression to be the decrease in plaque macrophages content. Such plaque regression has been achieved in mice by major reductions of plasma LDL-cholesterol (LDL-C) [4, 5]. In people, the reduction of LDL-C with rosuvastatin therapy, which increases the clearance of plasma LDL-C, led to a regression of coronary atherosclerosis as assessed by intravascular ultrasound, in the ASTEROID trial [6]. An alternative approach of lowering LDL-C is to reduce the production of the precursor of LDL particles, VLDL. Since microsomal triglyceride transfer protein (MTP) is essential for the assembly of VLDL [7], reduction of MTP expression or inhibition of this enzyme can reduce plasma VLDL and LDL. This important function of the MTP is used in the Reversa mouse model, which is a model for atherosclerosis regression. Reversa mice are deficient of LDLr, but also harbor additional genetic modifications (*Ldlr*^-/-^*Apob*^100/100^*Mttp*^fl/fl^*Mx1Cre*^+/+^) that allow the reversal of hyperlipidemia and atherosclerosis by conditional deletion of MTP [4, 8]. Similarly, we used the MTP inhibitor, BMS 212122, to induce regression of atherosclerosis in *LDLr-/-*mice [5].

Recently, a new model for the induction of atherosclerosis progression in mice was described [9, 10], circumventing the need for germ line-encoded models, such as low-density lipoprotein (LDL) receptor deficient mice (*LDLr-/-*) and apolipoprotein E deficient mice (*Apoe-/-*). By exploiting the functions of Proprotein convertase subtilisin/kexin type 9 (PCSK9), a protein that directs hepatic LDL receptors for degradation [11], it was shown that a single injection of recombinant adeno-associated virus (rAAV) encoding gain-of-function mutant forms of PCSK9, human PCSK9D374Y or mouse PCSK9D377Y (AAVmPCSK9), was sufficient to reduce LDL receptor expression, increase plasma LDL cholesterol and induce atherosclerosis in mice and hamsters [9, 10].

As many of the current mouse models for atherosclerosis regression are complicated by the requirement for genetic modifications, we sought to establish a model of atherosclerosis regression that is independent of the genotype of the mouse. Here, we show that atherosclerosis, induced by the injection of AAVmPCSK9 into wild-type (WT) mice that were fed with a western diet, could be reversed by a diet switch to chow, even without the treatment with an MTP inhibitor.

## Materials and methods

### Animals

This study was carried out in strict accordance with the recommendations in the Guide for the Care and Use of Laboratory Animals of the National Institutes of Health. The protocol was approved by the Committee on the Ethics of Animal Experiments of NYU (Permit Number: 150807–01). At the end of the experiments, all mice were sacrificed with a CO2 chamber. C57BL6/J male mice (The Jackson Laboratories, Bar Harbor, USA) were weaned at 4 weeks, injected intraperitoneally once with AAVmPCSK9 (AAV.8TBGmPCSK9D377Y, Penn Vector Core, using a plasmid kindly provided by Dr. Daniel J. Rader) at 5×10^11^ viral particles/mouse and placed onto a Western diet (Dyets Inc., Bethlehem, USA, Dyet #101977) for 16 weeks, and at this time point all the mice were transferred to a different room. Mice were then harvested for baseline analysis (base-line group), continued on Western diet (progression group), switched to a chow diet (chow regression group; this group served as a control for the diet change that was also performed in the MTP inhibitor group) or to a chow diet containing MTP inhibitor (BMS 212122; concentration: 25 mg per kg chow diet; kindly provided by Dr. David Gordon; BMS/Squibb) (MTPi group) for 6 weeks before harvesting (n = 9–11).

### Lipid and lipoprotein analysis

Plasma total cholesterol levels were determined by enzymatic assays (Wako Chemicals, Richmond, USA). For plasma lipoprotein analysis, equal volumes of plasma from each group were pooled. Lipoproteins were separated by fast-performance liquid chromatography (Superose 6 10/300 GL column, GE Healthcare, PA, USA) on a Shimadzu HPLC system. Following separation, fractions were collected and cholesterol content was quantified by colorimetric assays.

### Western blotting

For analysis of hepatic LDL receptor levels, liver tissue lysates were run in 4–20% Novex Tris-Glycine Gels (Invitrogen) under denaturing conditions and blots were stained with goat anti-LDL receptor (1:1000, Cat. # AF2255, R&D systems) and anti-α-tubulin antibodies (Sigma, clone T5168). Immunoreactive bands were visualized using the Odyssey imaging system (LI-COR Biosciences), by using fluorescent dye—labeled secondary Abs.

### Plasma PCSK9 ELISA

A mouse specific ELISA was used to measure the sum of D377Y-mPCSK9 and endogenous mPCSK9 (Biolegend cat. no. 443207).

### Determination of hematological parameters

A Genesis Hematology System (Oxford Science, Oxford, CT) was used to determine hematological parameters, including white blood cells (WBC), neutrophils, monocytes, lymphocytes, eosinophils, and basophils.

### Liver transaminases

Levels of liver transaminases (AST and ALT) were measured by ANTECH Diagnostics (Farmingdale, New York, USA).

### Histology and morphometrics

At harvesting, aortic roots were removed, perfused with 10% sucrose/saline and embedded in OCT. Serial sections were cut and stained for CD68 (AbD Serotec, Oxford, UK) as previously described [9]. Additional sections were stained with Oil Red O (Sigma). For collagen content, tissues were stained with picrosirius red and quantified using polarizing light microscopy. Intimal lesions and stained areas were quantified by computer-aided morphometric analysis of digitized images (ImagePro Plus 3.0 software, Silver Spring, USA). Lesion development in the aortic arch was determined using the en face technique. Briefly, the entire aorta was removed and cleaned for periadventitial fat, cut open longitudinally, and stained with Oil Red O (60% solution for 10 minutes at 37°C).

Steatohepatitis was assessed on paraffin embedded liver sections after hematoxylin and eosin (HE) staining.

### Statistical analysis

Data are expressed as mean ± SEM. Data were analyzed by one-way ANOVA with post hoc multiple comparison Tukey tests (Prism 6.0, GraphPad Software Inc., San Diego, USA). For comparisons between two groups at each time point the unpaired t-test was used; p < 0.05 was considered significant.

## Results

### AAVmPCSK9 injection induces hypercholesterolemia in mice

Liver transduction with an AAV vector expressing a gain-of-function mutation of PCSK9 under the control of the human α1-antitrypsin promoter induces stable changes in plasma lipid profile and atherosclerosis [9, 10]. Here, in order to establish an atherosclerosis regression model independent of genetic background, we utilized a similar virus, expressing murine PCSK9 D377Y, under the control of thyroid-binding globulin (TBG) promoter, which is a liver specific promoter.

**Fig 1:**
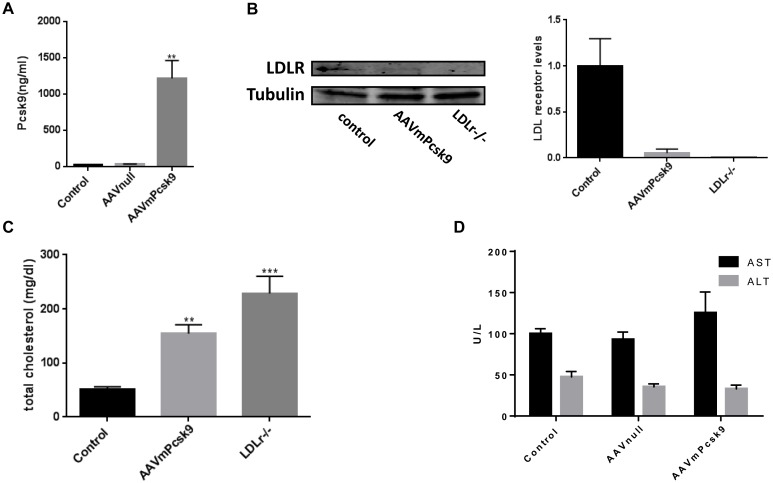
Hypercholesterolemia induced by recombinant Adeno-Associated Virus (rAAV)8-D377Y-murine Proprotein Convertase Subtilisin/Kexin type 9 (mPCSK9) in chow diet-fed mice. **A**. Serum levels of murine PCSK9 protein in mice injected with AAVmPCSK9 virus, AAV*null*–injected or saline-injected control mice. **P<0.01. **B**. Liver LDL receptor protein levels analyzed by western blot and normalized to α-tubulin (n = 3). **C**. Plasma total cholesterol of chow diet—fed mice. **P<0.01 compared with control, ***P<0.01 compared with control and AAVmPCSK9. **D**. Serum alanine aminotransferase (ALT) and aspartate aminotransferase (AST) were measured at the same time point and determined as described in Materials and Methods. Values are mean ± SEM; n = 3.

A single intra-peritoneal (i.p.) injection of 4-weeks-old wild-type C57BL/6J mice with 5×10^11^ viral particles/mouse resulted in a 50-fold increase in plasma PCSK9 levels, even at 22 weeks post injection (Fig 1A). As expected, the increase in PCSK9 resulted in a decrease in LDL receptor expression in the liver (Fig 1B) and an increase in plasma cholesterol levels in mice maintained on a regular chow diet (Fig 1C). At 22 weeks post injection, total plasma cholesterol in AAVmPCSK9 injected mice was 3 fold more than that in WT controls (154±16 versus 50±5 mg/dL) and similar results were observed 2 weeks post-injection (data not shown), demonstrating the chronic effect of a single AAV injection on systemic lipid levels.

Interestingly, the total cholesterol levels in (*LDLr-/-*) mice were even higher (228±31) than the AAVmPCSK9 injected mice, and this could be explained by the total deficiency of LDL receptor in the *LDLr-/-* mice, whereas in the AAVmPCSK9 injected mice the LDL receptor was still detectable (Fig 1B).

Hepatotoxicity could theoretically be a possible adverse effect of an AAV8 vector as it has strong liver tropism [12]; however, AAV viral infection does not elicit any reported adverse responses in animals, and post-injection levels of serum alanine aminotransferase (ALT) and aspartate aminotransferase (AST) were similar to those of uninjected mice or mice injected with control AAV8 viral particles (AAV-*Null*) (Fig 1D). Thus, AAV viral injection and expression of PCSK9D377Y does not induce hepatotoxicity in mice.

### Chow diet switch and MTPi can reverse hypercholesterolemia and hypercholesterolemia-associated monocytosis induced by AAVmPCSK9 injection

**Fig 2:**
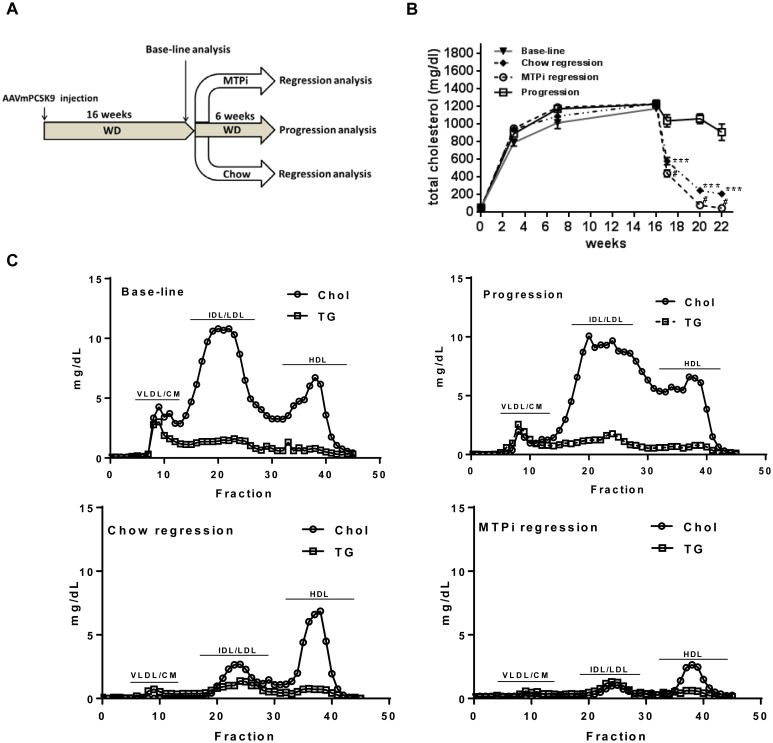
Hypercholesterolemia in AAVmPcsk9-injected, Western diet-fed mice can be reversed by a diet switch and MTPi treatment. **A**. Experimental design: C57BL/6J mice were i.p. injected once with AAVmPcsk9 and were placed on a Western diet for 16 weeks. At 16 weeks, the baseline group of mice was sacrificed and the remaining were continued with Western diet (progression group) or switched to chow (chow regression group) or MTPi diet (MTPi regression group), for another 6 weeks. **B**. Plasma levels (mg/dL) of total cholesterol in baseline, progression, chow regression and MTPi regression groups. ***P<0.001 when compared with base-line, # p<0.01 when compared with chow regression group. Values are mean±SEM; n = 9–11. **C**. Fast protein liquid chromatography (FPLC) profile of cholesterol (Chol) and triglyceride (TG) in pooled samples at the end of the experiment. CM/VLDL, chylomicron and very low-density lipoprotein; HDL, high-density lipoprotein; IDL/LDL, intermediate density lipoprotein.

To establish an atherosclerosis regression model using the AAVmPCSK9 vector, 4-week old C57BL/6J mice were injected i.p. with AAVmPCSK9, and were switched to Western diet. Following 16 weeks of Western diet, mice were divided to 4 groups: 1) base-line group, in which mice were sacrificed and analyzed for lipid plasma profile and atherosclerotic plaque size; 2) progression group, in which mice were continued on Western diet for an additional 6 weeks, and then analyzed similarly; 3) chow regression group, in which mice were switched to chow, and were analyzed 6 weeks later; 4) MTPi regression group, in which mice were switched to a chow diet containing MTPi, and were analyzed after 6 weeks (Fig 2A).

To achieve a maximal reduction in plasma lipids, the MTPi treatment was combined with a diet switch to a normal chow, since in a previous study, feeding hyperlipidemic *apoE-/-* mice with a western-type diet along with MTP inhibitor treatment maintained high plasma levels of cholesterol (500 mg/dl) and failed to result in regression of atherosclerosis [13]. In addition, we wished to simulate the clinical scenario, in which lipid lowering drugs are considered to be an adjunct to life style changes, a major one being the adoption of a healthier diet. Plasma total cholesterol level increased up to 941±103 mg/dl in 3 weeks post injection, and was stable at that level, until mice were switched to chow or to chow+MTPi at 16 weeks post injection, which resulted in a decrease in cholesterol level to 210±31 mg/dl and to 48±14 mg/dl, respectively, 6 weeks following diet switch (Fig 2B). As expected, diet change and MTPi treatment also resulted in weight loss (S2 Fig).

To assess the changes that occurred in the different cholesterol fractions, we analyzed cholesterol and triglyceride distribution in plasma samples by fast protein liquid chromatography (FPLC). The increases in total cholesterol and triglyceride in the AAVmPCSK9 injected mice that were fed a Western diet were mainly because of accumulation of LDL and intermediate density lipoprotein (IDL, Fig 2C), as expected from the reduction seen in LDL receptor expression (Fig 1B**)**. Once the mice were switched to chow or to chow+MTPi, there was a change in the distribution of the cholesterol fractions towards an increased concentration of HDL relative to LDL and VLDL, as previously demonstrated for WT mice on chow [14].

Hypercholesterolemia is also known to promote monocytosis [15]. Consistent with the normal cholesterol levels in the regression groups, we also noted an almost 50% decrease in blood monocytes counts in the regression groups compared with baseline and the progression groups, reaching statistical significance in the MTPi regression group (Table 1).

**Table 1:** White blood cells counts.

	Base-line	Progression	Chow regression	MTPi regression
**WBC 10^9/L**	2.72**±**1.075	2.576±1.35	2.04**±**1.11	2.35± 1.58
**NE 10^9/L**	0.80**±**0.36	0.694±0.33	0.52**±**0.34	0.86±1.14
**LY 10^9/L**	1.70**±**0.65	1.683±0.97	1.36**±**0.70	1.35± 0.52
MO 10^9/L*	0.13±0.06	0.137±0.06	0.09**±**0.07	0.08±0.03*
**EO 10^9/L**	0.06±0.081	0.039±0.04	0.04**±**0.05	0.03±0.04
**BA 10^9/L**	0.04±0.05	0.022±0.03	0.03**±**0.03	0.02± 0.03

* p-value<0.05 when compared with base-line or progression group.

Values are mean ± standard deviation; n = 9–11.

### MTPi treatment and chow diet switch can induce regression of atherosclerosis in AAVmPCSK9 injected mice without evidence of hepatic toxicity

Reduction of LDL cholesterol levels to near normal levels can induce regression of atherosclerosis [4, 5], thus we hypothesized that we will be able to observe atherosclerosis regression in AAVmPCSK9 injected mice upon diet switch/MTPi treatment.

**Fig 3:**
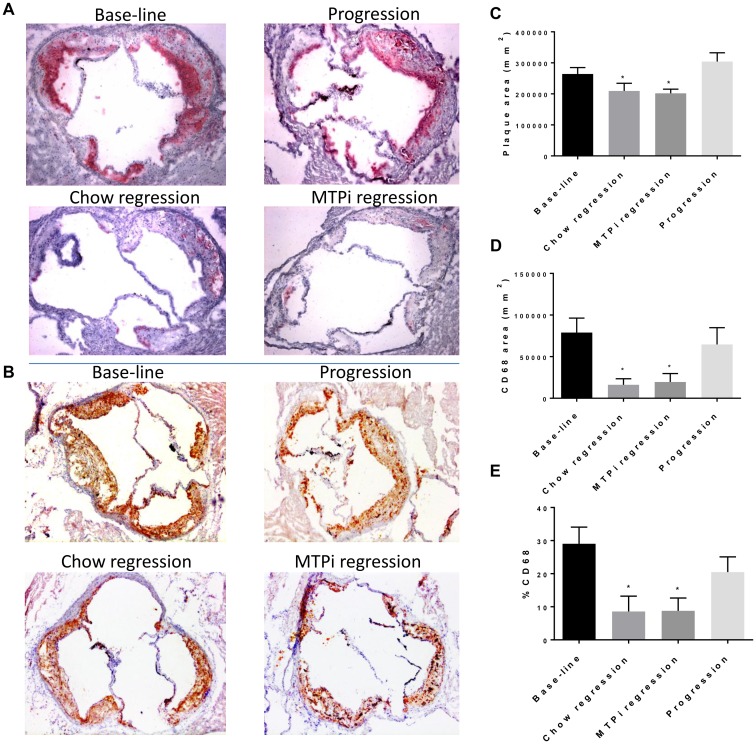
Regression of atherosclerosis induced by recombinant Adeno-Associated Virus (rAAV) 8-D377Y-murine Proprotein Convertase Subtilisin/Kexin type 9 (mPCSK9). **A**. Plaques of aortic root sections stained for macrophages (CD68) and (**B**.) Oil Red O from baseline, progression, chow regression and MTPi regression groups. (magnification ×10). **C**. Quantitative analysis of atherosclerotic lesion size in CD68–stained aortic sections, *p < 0.05 compared with progression group.** D**. Quantitative analysis of CD68-positive area in aortic sections. **E**. Quantitative analysis of CD68-positive area as percentage of plaque area, in aortic sections. *p < 0.05 compared with base-line group and progression group. Values are mean ± SEM; n = 5–9 in each group.

We found a significant reduction in plaque size and aortic lesions when comparing the regression groups with the progression group, from 264,323 ± 20,625 μm^2^ in the progression group to 209,457 ± 24,644 μm^2^ in chow regression group and to 201,821 ± 13,321 μm^2^ in the MTPi regression group (Fig 3A–3C, S1 Fig); However, there was no significant change in absolute plaque size when compared to baseline.

As alluded to earlier, plaque regression often is manifested not so much as a change in plaque size in mouse models, but in decreased macrophage content [16]. Presumably, plaque size is more modestly affected because the loss of MMP-secreting macrophages reduces collagen/ECM degradation. In the present studies, the plaque macrophage (CD68+) content and the percentage of CD68 positive area out of the total plaque area were both significantly decreased in the regression groups compared with either baseline or progression group (from 78,946 ± 17,322 μm^2^ and 64,659 ± 20,153 μm^2^ in baseline and progression groups respectively, to 16,220 ± 7242 μm^2^ and 19,511 ± 10,141 μm^2^ in chow regression and MTPi regression groups respectively; Fig 3D–3E). Indeed, an increase in collagen content was noticed in both regression groups compared with baseline and the progression groups (S3 Fig).

**Fig 4:**
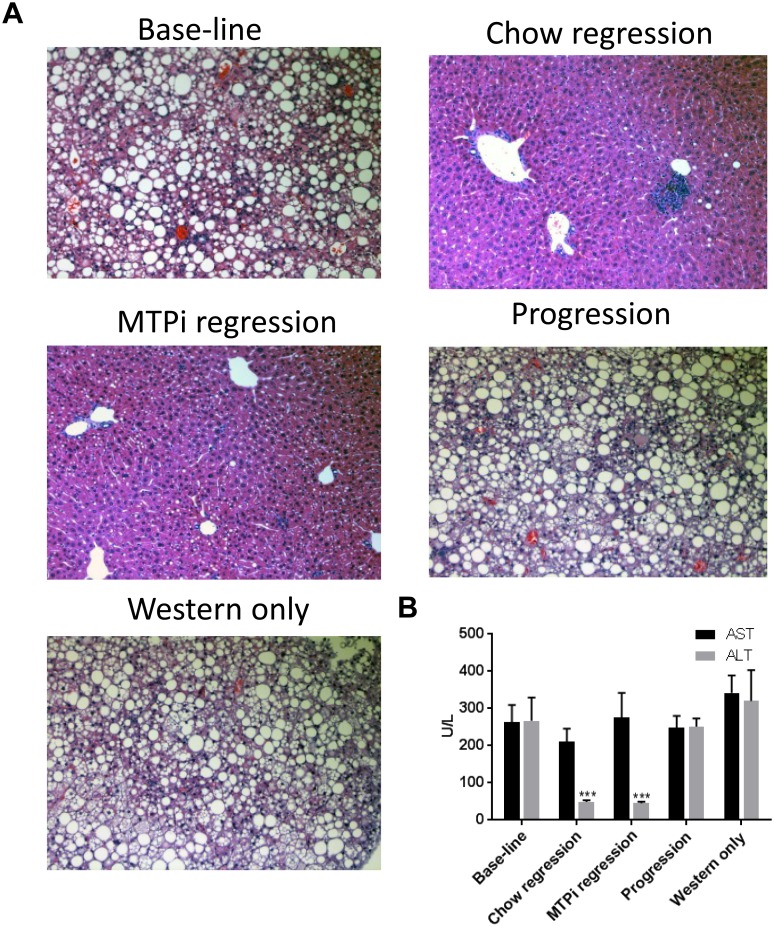
A. AAVmPCSK9 and MTPi do not contribute to liver toxicity induced by Western diet in mice. **A**. Representative H&E stained mouse liver sections of baseline, progression, chow regression and MTPi regression groups. “Western only” mice were saline-injected and Western diet-fed, serving as control for hepatotoxicity induced by Western diet alone. **B**. Plasma alanine aminotransferase (ALT) and aspartate aminotransferase (AST), were measured at the same time point and determined as described in Materials and Methods. *** p-value<0.001 compared with ALT values of baseline, progression and Western diet-only groups. Values are mean ± SEM; (n = 5).

Steatohepatitis can be induced by Western diet and MTPi [17, 18], therefore we used liver histological analysis and plasma liver enzymes to look for signs of steatohepatitis. Indeed, steatohepatitis was obvious in H&E sections of livers from baseline and progression groups, showing ballooning of hepatocytes, portal and intra-acinar inflammatory infiltrate, while minimal signs of steatohepatitis could be observed in the regression groups (Fig 4A). Importantly, the histological findings observed in the baseline and progression groups were also apparent in a Western diet-only control group (in which no virus was injected), implying that the majority of the steatosis seen in this model is induced by the Western diet itself and not by the injection of AAVmPCSK9. Moreover, the results imply that MTPi treatment is not a major cause of steatohepatitis under our experimental conditions, since there were no significant changes in the histological findings between the chow regression group and the MTPi regression group. In agreement with the histological findings, AST and ALT plasma levels were elevated in the baseline group and progression group to a similar level (AST in U/L: 264 ± 45, 248 ± 32; ALT in U/L: 267 ± 62, 251 ± 21, respectively, Fig 4B), similar to the Western diet-only control group (AST in U/L: 342 ± 47; ALT in U/L: 320 ± 83), while a clear reduction in ALT levels was observed in the two regression groups (chow regression—AST in U/L: 210 ± 35; ALT in U/L: 49 ± 5; MTPi regression—AST in U/L: 275 ± 67; ALT in U/L: 45 ± 4). In addition, there were no significant differences in AST and ALT plasma levels between the chow regression group and the MTPi regression group.

## Discussion

In the present study we found that reversal of AAVmPCSK9-induced hyperlipidemia by diet switch to chow, with or without treatment with an MTP inhibitor, leads to the regression of atherosclerosis as assessed by the percentage of macrophages in the plaques. This was definitely associated with the reduction in non-HDL-C plasma levels upon both treatments, in particular in the MTPi treated group. Interestingly, we have previously shown that a diet switch to chow without additional treatment is not enough to induce plaque regression in *Ldlr-/-* mice [5], while in the present study we could detect plaque regression in the chow diet switch-only group. It is important to note, however, that *Ldlr-/-* mice do not express any LDL receptor, while in the present model there is still detectable expression of the LDL receptor, that leads to total cholesterol plasma level that is lower than in *Ldlr-/-* mice on chow (~170 versus ~220 mg/dl on chow); Hence, we assume that the chow diet switch in the current model induces a more robust reduction in non-HDL-C levels, when compared with *Ldlr-/-* mice, and therefore permits plaque regression even with diet switch alone.

Consistent with the potent reduction of cholesterol in our model, we also noticed a reduction in monocytosis (Table 1), that is known to correlate with LDL cholesterol levels. While others also reported a trend towards neutrophilia in *Ldlr-/-* mice [15], albeit non-significant, we could not detect this in our model. Indeed, one could speculate that there might be additional, LDL-independent functions of PCSK9 in the bone marrow, that results in this discrepancy.

In the current model we noticed signs for steatohepatitis that could be attributed to the hyperlipidemia induced by western diet. Interestingly, upon diet switch to chow, with or without treatment with MTPi, we noticed a normalization of liver histology but a selective reduction of ALT versus AST. This consistent increase in AST could be related to some residual liver pathology, a longer time needed to resolve relative to ALT, or to the fact that AST has additional sources. In addition, AST and ALT can measure distinct aspects of hepatic inflammation; for example, when the inflammation in the liver progresses to fibrosis, ALT activity declines, and the ratio of AST to ALT increases [19]. Remarkably, despite a similar regression in atherosclerosis in the two regression groups, the cholesterol levels in the MTPi treated group were lower compared with the chow-only group; this minor discrepancy could possibly stem from the inherent variability that exists in current methods of plaque morphometric analysis, as opposed to the accurate methods for plasma cholesterol measurement, or that below a certain level of non-HDL-C, no further improvement occurred during the time frame of the study.

Several gain-of-function mutations in PCSK9 [20] can induce hypercholesterolemia and atherosclerosis [21, 22]. The mutation that we used to induce hypercholesterolemia in mice, analogous to amino-acid substitution of Asp374 by Tyr (D374Y) in humans [23, 24], increases the affinity of PCSK9 for the LDL receptor *in vitro* by ≥10-fold [25], and is the mutation described in PCSK9 with the greatest effect, resulting in cholesterol levels above 500 mg/dl in affected subjects. However, in spite of the potency of this mutant, when we targeted the assembly of VLDL and LDL, by using MTPi, there was a robust reduction in total cholesterol. We believe that this finding might have clinical significance to the people who harbor this mutation, and MTPi treatment, clinically approved, should be considered in this population.

In conclusion, the model presented here will be useful for testing genetic and pharmacologic approaches to regress atherosclerosis without the need for costly and time-consuming breeding with *ApoE* or *LDLr* deficient mice. We have assumed that lipid-lowering therapies will be standard of care in high risk populations, and the ability of a candidate factor to accelerate the regression process in this context will be easy to test. Also, by circumventing the need for extensive breeding programs in pre-clinical studies, the number of mice used for atherosclerosis regression research will be reduced.

## Supporting information

S1 Fig Regression of atherosclerosis as assessed by en-face lipid staining of aortae in mice made hypercholesterolemic by recombinant Adeno-Associated Virus (rAAV) 8-D377Y-murine Proprotein Convertase Subtilisin/Kexin type 9 (mPCSK9). Representative en face Oil Red O—staining of aortas from mice in the baseline, progression, chow regression and MTPi regression groups.
(TIF)

S2 Fig Body weight of mice. Measurements were done at the end of the experiment. ** p-value<0.01. Values are mean ± SEM; (n = 9–11).
(TIF)

S3 Fig Regression of atherosclerosis increases collagen percentage in the plaque. Picrosirius red staining (under white and polarized light) of collagen (magnification ×10) in the aortic roots are shown for each group. The areas of the plaques occupied by CD68+ cells and collagen (the latter as detected by polarized light) were quantified by Image Pro Plus Software and displayed in the graphs. Results are expressed as the percentage of plaque area. * p-value<0.05 compared with baseline and progression groups. Values are mean ± SEM.
(TIF)
