# Supplementary figures and images for: A wild-type mouse-based model for the regression of inflammation in atherosclerosis
Source: PLoS One. 2017 Mar 14;12(3):e0173975. doi: 10.1371/journal.pone.0173975 (PMC5349694; doi:10.1371/journal.pone.0173975)

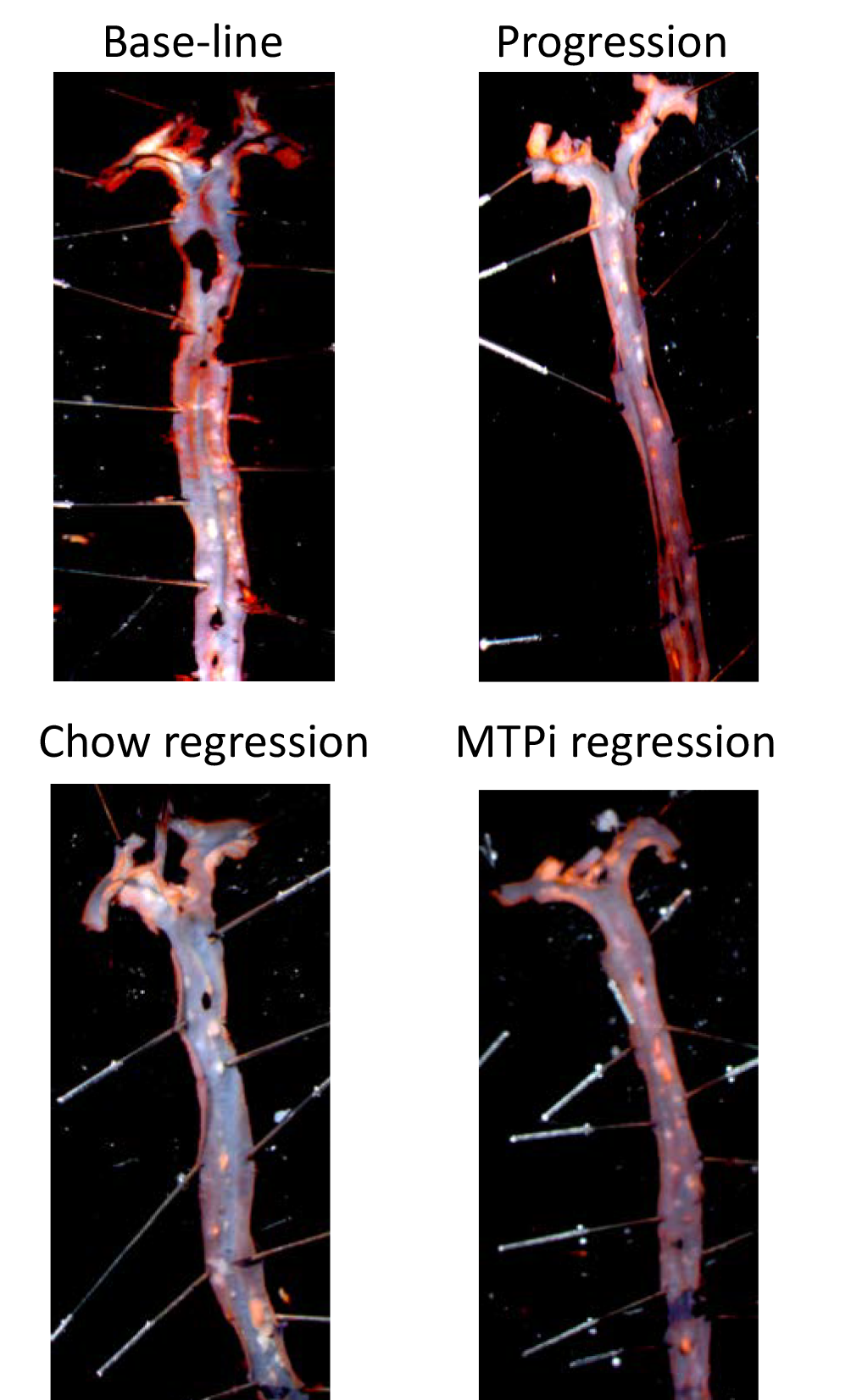

Supplement: S1 Fig — Representative en face Oil Red O—staining of aortas from mice in the baseline, progression, chow regression and MTPi regression groups. (TIF) [file pone.0173975.s001.tif]

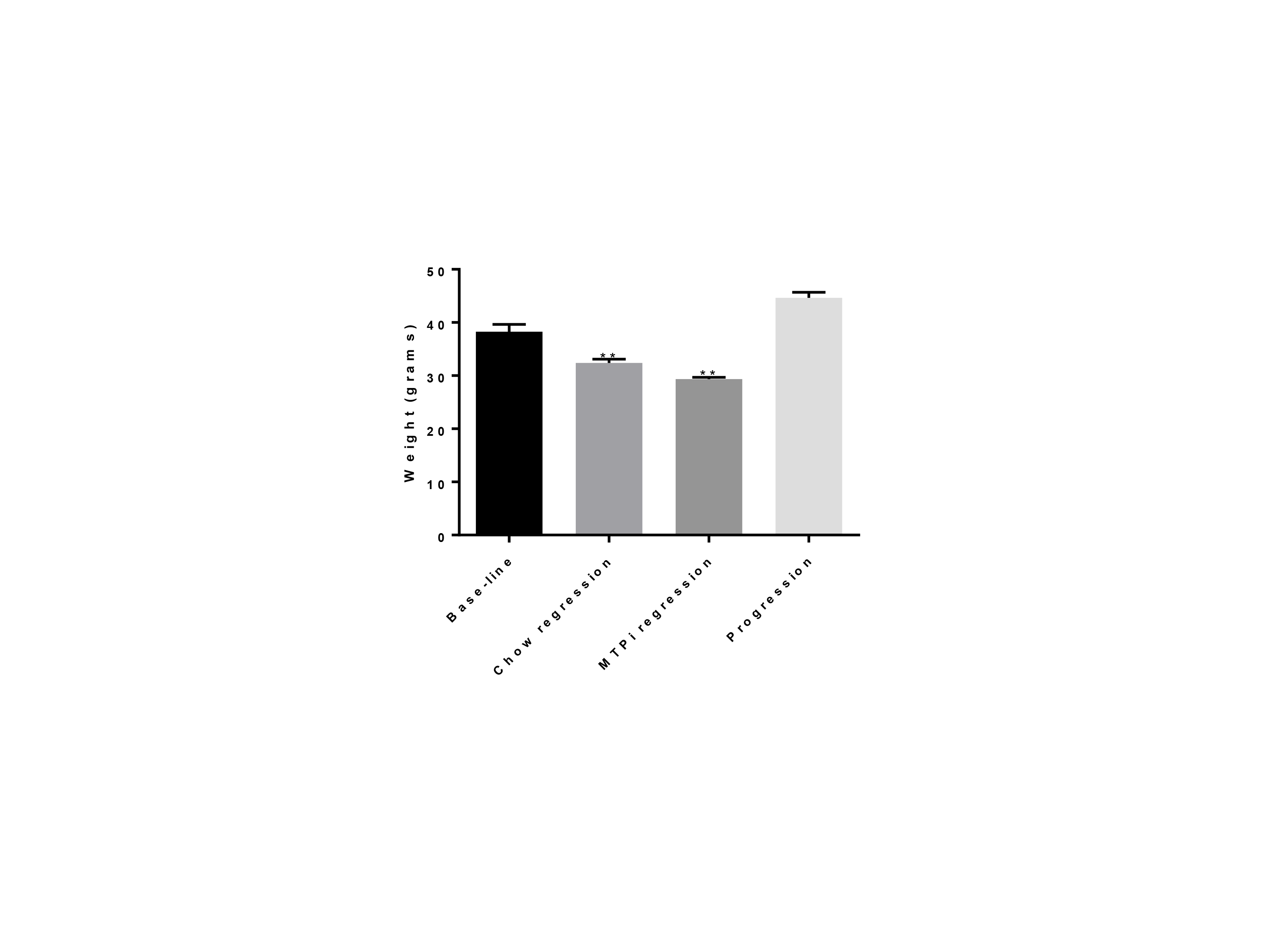

Supplement: S2 Fig — Measurements were done at the end of the experiment. ** p-value<0.01. Values are mean ± SEM; (n = 9–11). (TIF) [file pone.0173975.s002.tif]

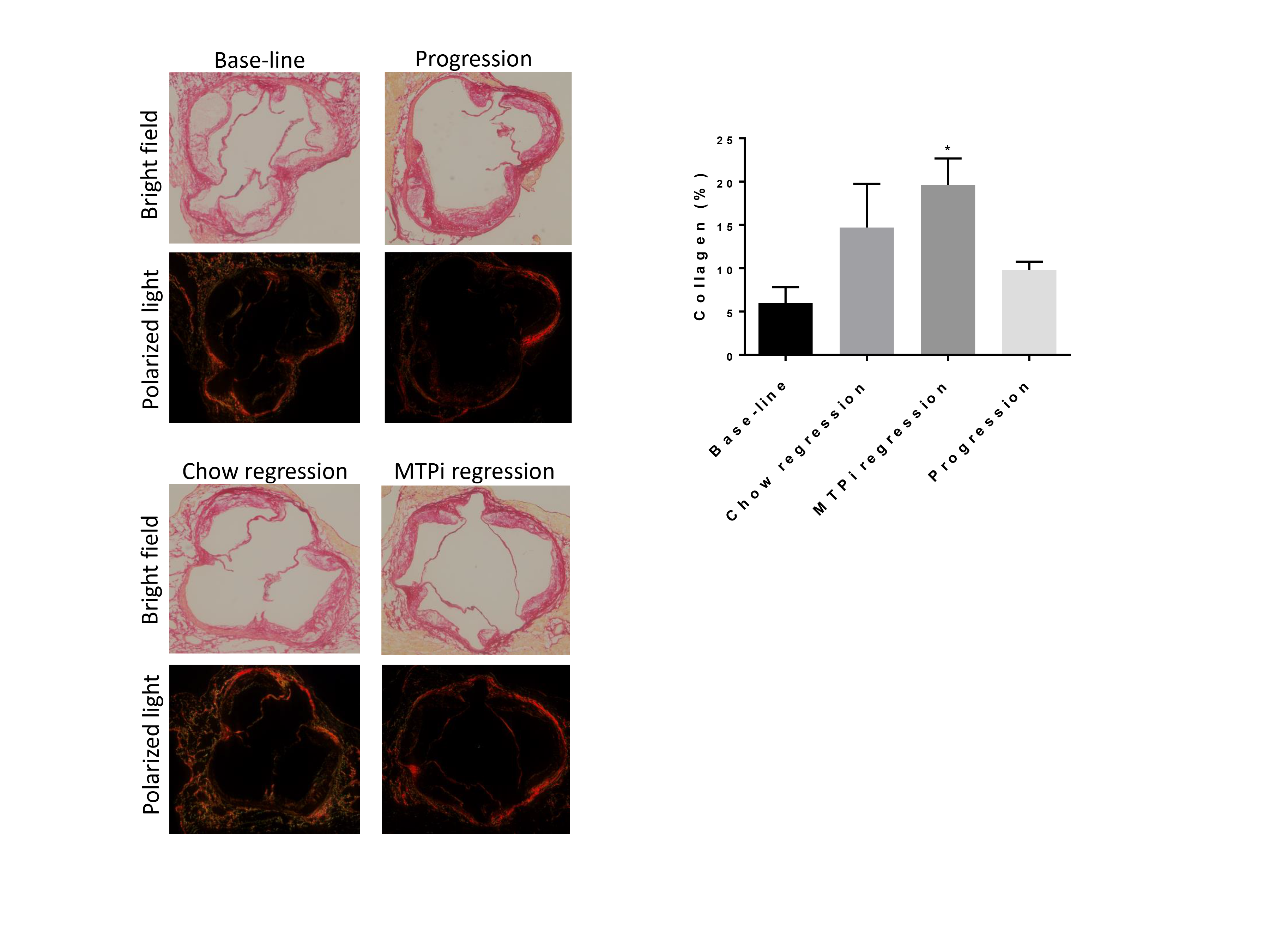

Supplement: S3 Fig — Picrosirius red staining (under white and polarized light) of collagen (magnification ×10) in the aortic roots are shown for each group. The areas of the plaques occupied by CD68+ cells and collagen (the latter as detected by polarized light) were quantified by Image Pro Plus Software and displayed in the graphs. Results are expressed as the percentage of plaque area. * p-value<0.05 compared with baseline and progression groups. Values are mean ± SEM. (TIF) [file pone.0173975.s003.tif]
